# Women’s Health and Gynecology: Old Challenges and New Insights

**DOI:** 10.3390/ijerph192416589

**Published:** 2022-12-09

**Authors:** Antonio Sarría-Santamera, Antonio Simone Laganà, Milan Terzic

**Affiliations:** 1 Department of Medicine, Nazarbayev University School of Medicine, Astana 010000, Kazakhstan; 2 Unit of Gynecologic Oncology, ARNAS “Civico—Di Cristina—Benfratelli”, Department of Health Promotion, Mother and Child Care, Internal Medicine and Medical Specialties (PROMISE), University of Palermo, 90127 Palermo, Italy; 3 Clinical Academic Department of Women’s Health, Corporate Fund “University Medical Centre”, Astana 010000, Kazakhstan; 4 Department of Obstetrics, Gynecology, and Reproductive Sciences, University of Pittsburgh School of Medicine, Pittsburgh, PA 15213, USA

The complexity of women’s health goes far beyond medical and surgical knowledge and the achievements of the clinical specialty of Obstetrics and Gynecology, spanning not just the research dimensions of molecular biology, genetics, epidemiology, or health services but also being influenced by gender, social, and psychological relevant factors. Those involved in and who are responsible for the clinical or health policy management of women’s health services should reflect on the findings to improve women’s quality of life and healthcare.

The objective of this editorial is to review issues relevant to women’s health that have been already published in the Special Issue. This Special Issue is not aimed to provide a comprehensive review of gynecological and obstetrical topics but rather seeks to reflect on contemporary issues that still require stronger scientific evidence in this clinical area. A variety of methodological approaches have been applied in the different papers published, reflecting the diversity of the topics investigated. The different scientific perspectives must be integrated and complemented to offer solutions to the challenges faced to provide the best care for women.

Leiomyomas are benign tumors derived from smooth muscle cells forming the myometrium and represent the most common benign female genital neoplasia. The etiopathogenesis of leiomyomas remains unclear, and the cluster of agonists and antagonists with their receptors involved in the development needs to be clarified. This Special Issue includes two papers that have provided valuable insights into leiomyomas.

Mayer–Rokitansky–Küster–Hauser (MRKH) syndrome is one of the most common causes of primary amenorrhea. This syndrome manifests as aplasia or hypoplasia of the uterus and the upper two-thirds of the vagina. The presence of leiomyomas in patients with MRKH syndrome is rare, but the presence of a gynecological complication should always be considered. Romano et al. [1] indicate that since the occurrence of new myomatous neoplasms in patients with MRKH, starting from uterine rudiments or not, could cause an acute abdomen requiring urgent surgery, patients who undergo pelvic surgery could benefit from the preventive remotion of uterine residues by minimally invasive surgery.

Cotyledonoid leiomyoma is an unusual uterine myoma due to some ultrasound features that mimic a malignant lesion that macroscopically resembles placental cotyledons, with large reddish and spongiform nodulations and often protruding into the pelvic cavity. Although the diagnosis may be entirely accidental, in some cases, it takes on extremely atypical features that may lead to the suspicion of malignancy, leading surgeons to opt for radical surgery. Therefore, Buonomo et al. [2] suggest that it is important to be aware of this unusual form of fibroid and its dissecting variant to avoid unnecessary extensive surgery. Typical ultrasound with magnetic resonance imaging features and the placenta-like appearance of the bulky mass during surgery may facilitate an appropriate diagnosis and surgical approach.

Pelvic floor disorders are also a common women’s health problem that affects their quality of life, having significant psychosocial effects, including low self-esteem, anxiety, frustration, and depression, with significant health, social, and economic burden associated with urinary incontinence and organ prolapse. Nevertheless, there is a concern that most maternity healthcare providers seldom discuss this issue with patients compared to other antenatal issues. The data from Nur Farihan and colleagues [3] suggest the need for healthcare providers to bridge the knowledge gaps, making pregnancy a great opportunity for educational interventions, avoiding women potentially not seeking medical attention and continuing to suffer in silence.

Providing effective alternatives for pelvic floor disorders is not simple. Different papers have investigated the efficacy of interventions aimed to improve pelvic floor disorders. Zhu et al. [4] will experimentally evaluate the effectiveness of the PELFLOW intervention, a general program for physical training of the pelvic floor via entire-body exercise that should be performed under the guidance of a professional physiotherapist. Gao et al. [5] are planning to conduct a trial to evaluate a video program designed for the prevention of pelvic floor problems developed through combining pelvic floor muscle training (PFMT) with global postural re-education. Hagovska et al., aim to establish the predictive value of pelvic floor muscle morphometry using 3D/4D ultrasound in relation to the success of PFMT [6]. Those papers explore different approaches but also diverse outcomes, probably reflecting the complexity of this problem as well as the lack of evidence regarding it.

Fertility is also a topic with significant social, clinical, and public health dimensions. The decline in birth rates has long concerned social, clinical, and public health scientists. The postponement of maternity, first defined as a “postponement transition” by Kohler et al. [7], arises from several interacting factors. One of the most important is the rising maternal age. Female reproductive efficiency is also directly and negatively affected by the age-dependent progressive decrease in ovarian reserve. The advent and diffusion of Assisted Reproductive Technologies (ART) may have led to a stronger belief that parenthood postponement lessens the biological limits of human reproduction. A lively debate has been triggered on so-called “social egg freezing” or on the possibility of the use of the cryopreservation technique. Planned Oocyte Cryopreserving (POC) is an alternative favored by many women. Cremonese et al. [8] provide in their paper the Italian perspective on POC, discussing policy, clinical, and ethical issues.

Gullo et al. [9] suggest that including the gender dimension throughout the diagnostic–therapeutic journey of infertile couples helps eliminate gender bias, allowing for equitable access to the design of infertility diagnosis and treatment. Owing to social constructs, female factors are more likely to seek medical attention, while male factors are not systematically analyzed and are overlooked: the male partners should be carefully managed by considering not only their sperm concentration, motility, and morphology, but also genetic tests and testicular histology and other psychological aspects to offer a tailored treatment and targeted use of ART.

Premature ovarian insufficiency (POI) is a clinical syndrome defined by the premature loss of ovarian activity, leading to a chronic hypo-estrogenic state in women under the age of 40 years. Tong et al. [10] map the knowledge structure and themes trends of POI therapy. Their findings confirmed that the hotspots of POI therapy are hormone replacement therapy and fertility preservation. Innovative techniques and novel therapeutic strategies for fertility preservation are attracting increased attention, providing a valuable reference for researchers to understand the further diagnosis and therapy for POI.

Polycystic ovary syndrome (PCOS) is among the most common endocrine disorders and a major cause of anovulatory infertility in women of reproductive age [11]. Multiple genetic and environmental factors play an important role in the occurrence of PCOS [12]. The consequences of this multifaceted disorder extend beyond the reproductive system affecting the metabolic, cardiovascular, immune, and psychological health of affected women. PCOS is characterized by various symptoms, including irregular menses and hirsutism, and it can also lead to infertility. Women diagnosed with PCOS are more likely to develop diabetes mellitus, obstructive sleep apnea, obesity, and depression.

Mir et al.’s [13] results demonstrated significant biochemical alterations in PCOS patients, including fasting glucose, free insulin, HOMA-IR, LDL, HDL, cholesterol, and hormones such as FSH, LH, testosterone, and progesterone. Their genetic analysis identifies that for estrogen receptor-α (ESR1 PvuII-rs2234693 T > C), the frequency of the T allele (fT) was significantly higher among patients with PCOS, while the frequency of the C allele (fC) was lower in patients (0.36 vs. 0.56) compared to controls. Their study also found a strong association of miRNA-146a (rs2910164 C > G) gene polymorphism with an enhanced risk of PCOS. The frequency of the C allele (fC) was also significantly higher. The frequency of the G allele (fG) was found to be lower in patients. The codominant, dominant and recessive models display a statistically significant association of polymorphic variations with PCOS. Moreover, the G allele was strongly associated strongly with PCOS susceptibility.

Endometriosis is also a complex condition with still significant clinical and epidemiological controversy, whose incidence may be estimated to range from 1.36–3.53 per 1000 Person Year [14]. This significant variability may not only be due to methodological issues and the specific limitations of the different designs and data analyzed, including case definitions and subject selection strategies, but also to the inherent heterogeneity of endometriosis. Catalan et al. [15] estimated an incidence of 1.11 per 1000, with a maximum of 1.60 in the 31–35-year-old age group, with a very strong north–south spatial gradient in a more industrialized and polluted area.

Pre-eclampsia is another complex, heterogeneous, multisystem disorder that represents a serious threat to maternal and fetal safety, being a leading cause of maternal mortality [16]. Pre-eclampsia is considered primarily a placental disorder and is part of the spectrum of “great obstetrical syndromes” that share a common pathophysiology centered upon disordered placentation [17]. Matrix metalloproteinases (MMPs) are a group of important regulators of angiogenesis and uterine remodeling that are involved in blastocyst implantation, spiral artery remodeling, and placenta formation. MMPs have been suggested to be associated with the development of pre-eclampsia. Bahabayi et al. [18] revealed that serum levels of MMP-2, -7, and -9 were significantly different between the pre-eclampsia and control groups during pregnancy, suggesting that increases in MMP-2 and MMP-7 levels and a decreased MMP-9 level seemed to be related to the pathogenesis of pre-eclampsia.

Pokorska-Niewiada et al. [19] identified that the relationship between the level of trace elements (namely Zn, Ni, Fe, Mn, Cu, Mg) and the level of hormones suggests that, in obese women with PCOS, nickel may play a role in inhibiting the processes of folliculogenesis and ovulation. Trace elements have been proven to have a strong influence on the normal metabolism of the body through interaction with many enzymes and hormones and play an important role in the proper course of ovulation. Disturbances in the concentration of trace elements in the body may contribute to endocrine disorders. Trace elements catalyze oxidative stress reactions and may lead to the formation of oxygen free radicals. However, it is still unclear whether this process is the cause or the effect.

Cervical cancer continues to be one of the most prevalent malignancies in women worldwide: to date, more than 90% of cervical cancer cases are caused by HPV infection. Screening and vaccination against HPV are reducing cervical cancer mortality, but environmental factors may also play a significant part in the disease’s progression. Sasivimolrattana and colleagues [20] investigated the bacterial, fungal, and viral communities in the cervix of patients infected with HPV16 and Hr-HPV at various precancerous stages. Patients infected with HPV could be divided into cases who are Lactobacilli-dominated (LD) and non-Lactobacilli-dominated (NLD), indicating that Lactobacillus spp. may play a significant role in bacterial diversity, while HPV infection played a crucial role in both bacterial and human viral diversity. However, the mode of transmission and function in the pathogenesis of many microorganisms, particularly viruses, remain obscure and require further investigation.

Low- and middle-income countries (LMIC) continue to show too-high rates of under-screened women. Self-sampling methods, such as vaginal self-sampling and urine sampling for HPV diagnosis, have demonstrated high acceptability and sensitivity for cervical cancer screening; they are less invasive and could be more attractive to increase the uptake in under-screened and never-screened women and overcome barriers at different levels of interaction. Vega-Crespo [21] has shown that vaginal and urine self-sampling methods have a high diagnostic capacity and similar sensitivity and specificity compared with clinician sampling for the diagnosis of HPV, with a satisfactory correlation between HPV genotypes among the three tests. Zhang et al. [22] have identified how educational interventions may be successful in rural, vulnerable populations with limited access to healthcare to effectively promote the uptake of cervical cancer screening: their findings indicate the need to develop cervical cancer screening-related health education and theory-driven, community-involved, group-based, and healthcare professional-led educational approaches. In terms of the content of cervical cancer screening education, tailored educational materials have to take into account the influence of sociocultural barriers on behavior in rural areas.

Women with a cervical cancer diagnosis experience important healthcare informational challenge. There is strong evidence indicating that hierarchical medical encounters inhibit women from expressing their doubts and concerns [23], limiting women’s capacity of acquiring knowledge about their condition as well as the opportunities for self-care or adherence to medical recommendations, while increasing anxiety and fear of cancer, making it difficult for women to understand and effectively participate in decision making. Freijomil-Vazquez et al. [24] report that gendered relations in cervical cancer medical encounters are based on hidden, judgmental moral assumptions, making women feel irresponsible and blamed for contracting the human papillomavirus infection. Healthcare providers must consider women’s non-biomedical rationalities, self-care skills, and the need to respect their decisions. Healthcare should be offered to both women and their partners (who may be male, female, gender non-conforming, cis, or transgender) to reduce the negative personal impact of being solely responsible for the management of cervical cancer.

Stabile et al. [25] report a case of vaginal perforation with organ evisceration after consensual sexual intercourse and performed a systematic review of vaginal perforations with or without evisceration in women. As well as the need for detailed anamnesis, checking the patient’s vital signs, and prompt vaginal and rectal examination to identify the site and characteristics of the lesion, it is very important to reduce the patient’s embarrassment and, most importantly, to understand if the patient has been a sexual victim. Appropriate wound healing will require avoiding potentially risky sexual behaviors and psychological support, so creating an appropriate patient–professional partnership appears critical.

Quality of life (QoL) involves a person’s physical health, psychological state, degree of independence, social relationship, personal beliefs, and environment. The relevance of assessing QoL is being recognized as critical for healthcare improvement. Women who are survivors from gynecological cancers suffer from a reduced QoL, including reduced psychological, social, and physical well-being. Hasan et al. [26] found that family caregivers of women with gynecological cancers also suffer from significantly reduced physical and psychological health. When a family member is diagnosed as suffering from cancer, there will be role changes and changes in family members’ expectations and responsibilities, which may have labor and financial consequences.

Healthcare management of complex diseases, such as most gynecological and obstetric diseases, is becoming more intricate and therefore must be based on the best evidence, with the highest standards in quality of care and patient safety and aim to maximize patients’ value and expectations [27]. Maes-Carballo et al. [28] report significant heterogeneity in the quality-of-care indicators proposed to assess the quality of care in breast cancer, making comparisons of results across populations or hospitals difficult, and highlighting relevant discrepancies among the studied integrated healthcare processes and clinical pathways. Quality indicators should be updated as medical care technology provides new alternatives for the care of patients with complex diseases, such as breast cancer.

The digitalization of health services has reached a new level. Digital technologies will become irreplaceable tools in healthcare. The Internet of Things, Big Data, Machine Learning, and Artificial Intelligence are changing the delivery of health services, providing faster decision-making in-patient diagnosis, treatment, and day-to-day monitoring, which can be especially valuable when healthcare professionals and systems experience extremely high workloads [29]. Sakko and colleagues [30] used the data available from the Unified Nationwide Electronic Health System (UNEHS) from the Republic of Kazakhstan during the period of 2014–2019 to investigate the prevalence, indications, and outcomes of the most common major gynecological surgeries by analyzing large-scale healthcare data and to identify possible opportunities for improvement in the local public health and clinical practice.

Women have unique health needs, and most diseases and conditions affect women differently than men. Women may experience health disparities throughout their lifespans because of their gender, historic health inequities in the healthcare system, and socioeconomic conditions. This Special Issue highlights relevant conditions that affect women’s health with papers that focus on molecular biology, genetics, clinical trials, epidemiology, health services research, or qualitative methods, exploring how to improve our scientific knowledge to improve women’s health and developing high-performing gynecology and obstetrical care.
